# Supplementary material for: Postoperative Changes in Pulmonary Function after Valve Surgery: Oxygenation Index Early after Cardiopulmonary Is a Predictor of Postoperative Course
Source: J Clin Med. 2021 Jul 23;10(15):3262. doi: 10.3390/jcm10153262 (PMC8348833; doi:10.3390/jcm10153262)
Supplement: Supplementary file 1 [file jcm-10-03262-s001.zip › jcm-1296799-supplementary.pdf]

Journal of
*Clinical Medicine*

MDPI

## Supplemental Materials

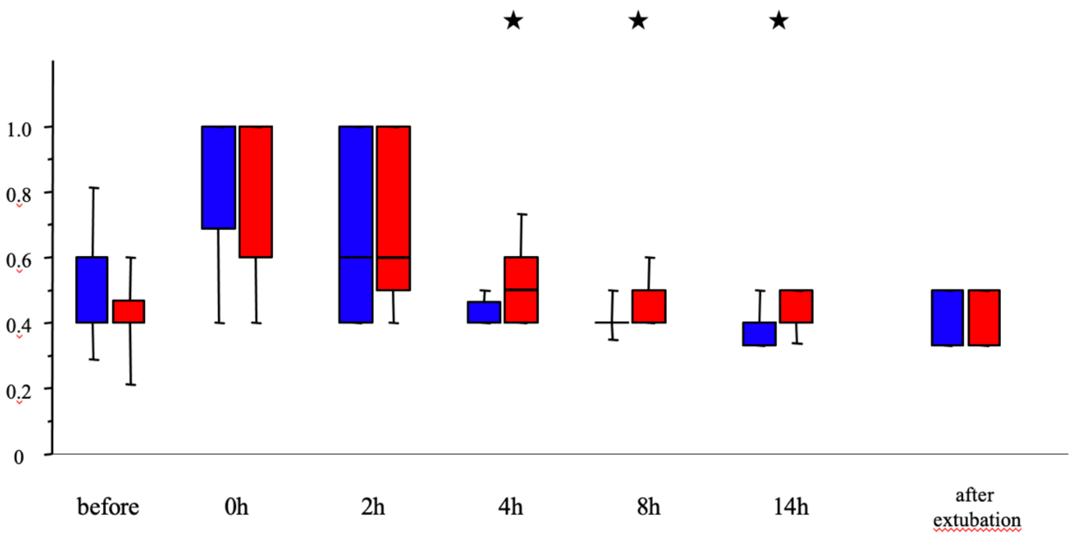


**Figure S1.** Time-dependent changes in FiO2. Panel A: Time-dependent change in FiO2 in patients with and without post-cardiopulmonary bypass (CPB) lung injury (PCLI). Both groups showed similar changes, but FiO2 in patients with PCLI was significantly higher than FiO2 in patients without PCLI at 4, 8, and 14 hours after CPB. Panel B: Time-dependent change in FiO2 in obese and non-obese patients. Both groups showed similar changes, but FiO2 in obese patients was significantly higher than FiO2 in non-obese patients at 4 and 14 hours after CPB.

*, $p < 0.05$ between the two groups.

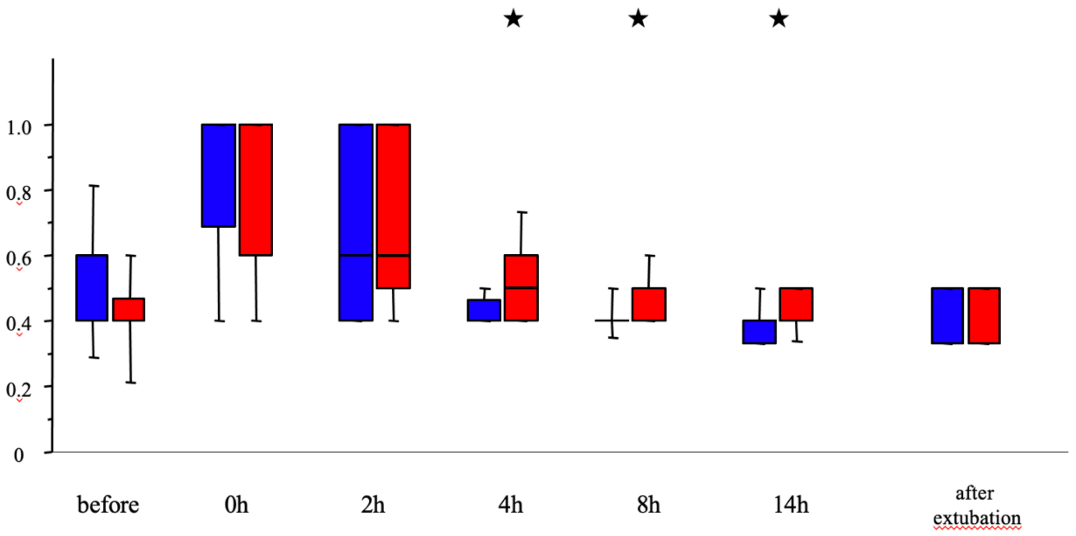


**Figure S2.** Time-dependent changes of PaCO2 in patients with and without post-cardiopulmonary bypass lung injury (Panel A) and those with and without obesity (body mass index > 25) (Panel B). In both Panel A and Panel B, PaCO2 levels were different between the two groups at some time points; however, PaCO2 was mostly within satisfactory range throughout the observation period.

*1, $p < 0.05$ vs. the value before surgery; *2, $p < 0.05$ between two groups.

**Supplemental Table S1**: Univariate and multivariate analyses for prolonged ventilation

| Univariate | P Value | Hazard Ratio | 95% CI |
|---|---|---|---|
| Ventilation≥24 hours | | | |
| *(preoperative factors)* | | | |
| Age | 0.156 | 0.980 | 0.953-1.008 |
| Male | 0.100 | 0.512 | 0.231-1.136 |
| Smoking history | 0.669 | 0.864 | 0.443-1.687 |
| Current smoking | 0.740 | 0.857 | 0.345-2.129 |
| Diabetes Mellitus | 0.045 | 0.476 | 0.231-0.982 |
| Hyperlipidemia | 0.782 | 1.100 | 0.560-2.160 |
| Hypertension | 0.015 | 0.263 | 0.090-0.775 |
| eGFR*[1] | <0.001 | 1.026 | 1.012-1.040 |
| COPD*[2] | 0.801 | 1.100 | 0.524-2.307 |
| PO2*[3] | 0.172 | 1.019 | 0.992-1.046 |
| PCO2*[3] | 0.521 | 1.022 | 0.956-1.093 |
| Previous stroke | 0.817 | 0.892 | 0.339-2.346 |
| NYHA≥II | 0.465 | 1.249 | 0.687-2.273 |
| LVEF | 0.016 | 1.028 | 1.005-1.052 |
| Pulmonary hypertension*[4] | <0.001 | 0.263 | 0.128-0.543 |
| Peripheral arterial disease | 0.002 | 0.145 | 0.044-0.483 |
| Emergency | <0.001 | 0.093 | 0.030-0.290 |
| Re-sternotomy | 0.250 | 0.344 | 0.056-2.123 |
| Euroscore-II | <0.001 | 0.850 | 0.777-0.930 |
| *(operative factors)* | | | |
| Aortic valve surgery | 0.444 | 1.300 | 0.664-2.546 |
| Mitral valve surgery | 0.645 | 1.173 | 0.595-2.311 |
| Aortic+Mitral valve | 0.050 | 0.393 | 0.155-0.998 |
| Additional procedures | 0.067 | 0.524 | 0.263-1.046 |
| Use of IABP | 0.002 | 0.261 | 0.110-0.621 |
| Fentanyl | 0.564 | 1.013 | 0.970-1.057 |
| Water balance | <0.001 | 1.000 | 0.999-1.000 |
| Lowest hemoglobin*[5] | 0.090 | 1.293 | 0.961-1.739 |
| Transfusion*[6] | <0.001 | 0.088 | 0.033-0.237 |
| Inotrope ≥ medium *[7] | 0.008 | 0.355 | 0.165-0.761 |
| Operation time | <0.001 | 0.989 | 0.986-0.993 |
| CPB*[8] time | <0.001 | 0.986 | 0.981-0.991 |

| | | | |
|---|---|---|---|
| ACC*9 time | 0.002 | 0.986 | 0.977-0.995 |
| PCLI*10 | <0.001 | 0.122 | 0.048-0.314 |
| Multivariate | P Value | Hazard Ratio | 95% CI |
| Ventilation≥24 hours | | | |
| DM | 0.163 | 0.356 | 0.083-1.522 |
| HT | 0.298 | 0.433 | 0.089-2.095 |
| eGFR | 0.655 | 1.006 | 0.981-1.031 |
| LVEF | 0.336 | 1.025 | 0.975-1.077 |
| Pulmonary hypertension | 0.521 | 0.615 | 0.139-2.718 |
| Emergency | 0.999 | <0.001 | 0.000-0.000 |
| Peripheral arterial disease | 0.679 | 0.584 | 0.046-7.449 |
| Euroscore-II | 0.614 | 0.957 | 0.808-1.134 |
| Use of IABP | 0.999 | 3677433012 | 0.000-0.000 |
| Water balance | 0.569 | 1.000 | 1.000-1.000 |
| Transfusion | 0.004 | 0.109 | 0.024-0.449 |
| Inotrope ≥ medium | 0.145 | 3.753 | 0.633-22.248 |
| Operation time | 0.093 | 0.990 | 0.979-1.002 |
| CPB time | 0.170 | 0.134 | 0.034-0.538 |
| ACC time | 0.806 | 0.997 | 0.977-1.019 |
| PCLI | 0.005 | 0.107 | 0.023-0.506 |

*1: estimated glomerular filtration rate. *2: chronic obstructive pulmonary disease. *3: values are under room air. *4: diagnosed in preoperative echo cardiogram or catheter examinations. *5: the lowest value during cardiopulmonary bypass. *6: transfusion of red blood cells > 4 units or frozen fraction of plasma >4 units or platelets during surgery. *7: dopamine + dobutamine ≥ 5 μg/kg/min or norepinephrine ≥ 0.15 μg/kg/min. *8: cardiopulmonary bypass. *9: aortic cross-clamp. *10: post-cardiopulmonary bypass lung injury.
